# Chemotactic Activity of Cyclophilin A in the Skin Mucus of Yellow Catfish (*Pelteobagrus fulvidraco*) and Its Active Site for Chemotaxis

**DOI:** 10.3390/ijms17091422

**Published:** 2016-08-29

**Authors:** Farman Ullah Dawar, Jiagang Tu, Yang Xiong, Jiangfeng Lan, Xing Xing Dong, Xiaoling Liu, Muhammad Nasir Khan Khattak, Jie Mei, Li Lin

**Affiliations:** 1 College of Fisheries, Huazhong Agricultural University, Wuhan 430070, China; farmandawar2012@yahoo.com (F.U.D.); tujiagang@mail.hzau.edu.cn (J.T.); 13697134024@163.com (Y.X.); lanjiangfeng@mail.hzau.edu.cn (J.L.); dongxingxinghg@163.com (X.X.D.); liuxl@mail.hzau.edu.cn (X.L.);; 2 Freshwater Aquaculture Collaborative Innovation Center of Hubei Province, Wuhan 430070, China; 3 Department of Zoology, Hazara University, Mansehra 21300, Pakistan; mnasir43663@googlemail.com

**Keywords:** yellow catfish, skin mucus, cyclophilin A, chemotaxis, immune response

## Abstract

Fish skin mucus is a dynamic barrier for invading pathogens with a variety of anti-microbial enzymes, including cyclophilin A (CypA), a multi-functional protein with peptidyl-prolyl *cis*/*trans* isomerase (PPIase) activity. Beside various other immunological functions, CypA induces leucocytes migration in vitro in teleost. In the current study, we have discovered several novel immune-relevant proteins in yellow catfish skin mucus by mass spectrometry (MS). The CypA present among them was further detected by Western blot. Moreover, the CypA present in the skin mucus displayed strong chemotactic activity for yellow catfish leucocytes. Interestingly, asparagine (like arginine in mammals) at position 69 was the critical site in yellow catfish CypA involved in leucocyte attraction. These novel efforts do not only highlight the enzymatic texture of skin mucus, but signify CypA to be targeted for anti-inflammatory therapeutics.

## 1. Introduction

The fish mucosal surface, like skin, is regularly exposed to pathogens and secretes adherent mucus that covers its epithelial layer [1,2]. Fish skin mucus is a natural, semipermeable, biochemically-diverse and dynamic barrier that is important for maintaining normal fish physiology and conferring defense against microbial infections [3,4]. Skin mucus characterizations from different fish species revealed several proteases, including serine and metalloproteases with strong antimicrobial capabilities [5,6,7]. Studies reported several innate immune factors, such as glycoproteins, lysozyme, antimicrobial peptides and immunoglobulins in fish skin mucus [8,9,10]. Others reported that fish skin mucus contains peroxidase, alkaline phosphatase, esterases, proteases, antiproteases [11], galectin-1, mannan binding lectin (MBL), serpins, cystatin B, FK-506 binding protein, proteasome subunits (α-3 and -7), ubiquitin, g-type lysozyme and CypA, as well [12].

CypA is an immunophilin with peptidyl-prolyl *cis*/*trans* isomerase (PPIase) activity and acts as a cellular receptor for immunosuppressive drug cyclosporine A (CsA). It regulates numerous cellular functions being a cytosolic protein; however, it is also considered as a critical immune mediator with strong anti-viral activity [13]. Furthermore, it is also involved in protein folding and trafficking [14], gene regulation [15], genome degradation [16] and cell signaling pathways, such as interleukin-2 tyrosine kinase (Itk) [17]. Extracellular CypA works against inflammatory stimuli, such as infection, oxidative stress and hypoxia [18,19,20]. Its association with different diseases, likes sepsis, asthma, periodontitis, aging, cardiovascular diseases, neurodegeneration, cancer and rheumatoid arthritis (RA), is widely evidenced [14,21]. CypA involvement is essential in the progression and inhibition of several viral infections and is widely targeted for controlling viral diseases [13]. CypA also functions against lipopolysaccharides (LPS) and bacterial challenge [22,23,24].

In addition to various physiological and pathological functions [25,26], extracellular CypA is a potent chemoattractant to induce human leucocytes migration during inflammation [27]. Cells secrete CypA in response to inflammatory stimuli and cell death [18], where it interacts with CD147, the main receptor for CypA on the cell membrane of human leucocytes, and exhibits leucocyte chemotaxis [26,28]. In rheumatoid arthritis (RA) patients, the abundant CypA upregulates the adhesion and invasion of neutrophils by direct binding to CD147 and subsequently destroys cartilage and bones [29]. In mouse, CypA interacts with typical chemokine (MIP-2) and increases leucocyte recruitment after acute lung inflammation [30]. In cattle, extracellular CypA was observed in tissues with inflammation and had shown a strong chemotactic activity for bovine peripheral blood cells [31]. Guo [32] demonstrated that CypA induced cell migration via CD147, which correlates with cell aggregation in Jurkat T cells. Similarly, Yeh [33] reported that channel catfish CypA has strong chemotactic activity for peritoneal macrophages. Recently, CypA was characterized from yellow catfish, with chemotactic activity for leucocytes [34]. However, its presence and function in yellow catfish skin mucus is unknown. Therefore, in the present study, first we have screened the skin mucus of yellow catfish and found defensive enzymes, including CypA. Furthermore, the CypA showed chemotactic activity for leucocytes in the skin mucus of yellow catfish. Additionally, a required critical site of yellow catfish CypA responsible for chemotaxis has also been discovered. This study does not only highlight the mucosal function of CypA, but would help to investigate this protein in the teleost immune and pathogenic processes in the future.

## 2. Results

### 2.1. Mass Spectrometry and Western Blot Analysis

**Figure 1:**
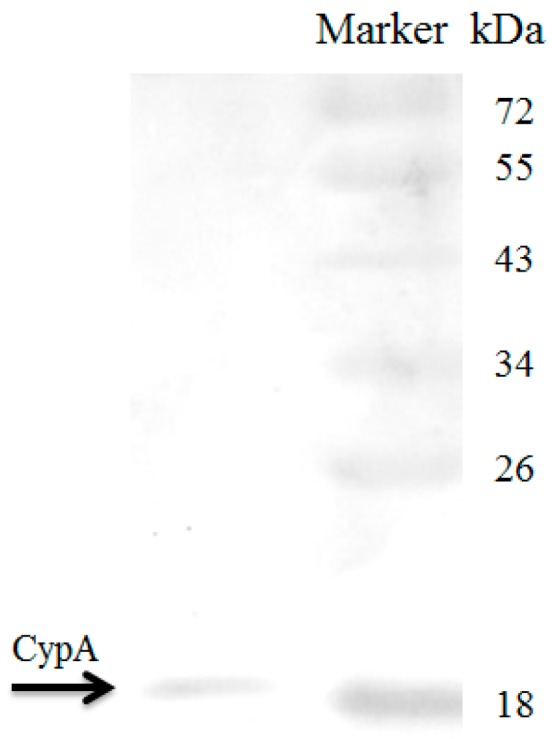
Result of CypA in the skin mucus detected by Western blot. Anti-CypA antibody was used to detect CypA. The CypA band is marked and equal to the precise size (~18 kDa) of the marker.

The mass spectrometric analysis identified and characterized the immune-relevant proteins in the skin mucus as interferon-induced GTP-binding protein Mx1, vasa short form, β-enolase, recombination activating protein 1, lsm12-like protein A and CypA. These peptides have shown hits to expressed sequence tags (EST)-bases, and their theoretical molecular masses (Da) were 72,882, 70,798, 47,773, 46,987, 21,644 and 17,724, respectively. All of the proteins are presented with their respective species and accession number to which they matched with the annotated score of 30, 31, 36, 28, 31 and 32, respectively (Table 1). The matching amino acid sequences, reference numbers and percent identity are shown in supplementary materials (Table S1). CypA was detected by Western blot in the skin mucus with a precise band size as that in other body tissues and is shown in Figure 1.

### 2.2. Chemotaxis Assay of Skin Mucus

**Figure 2:**
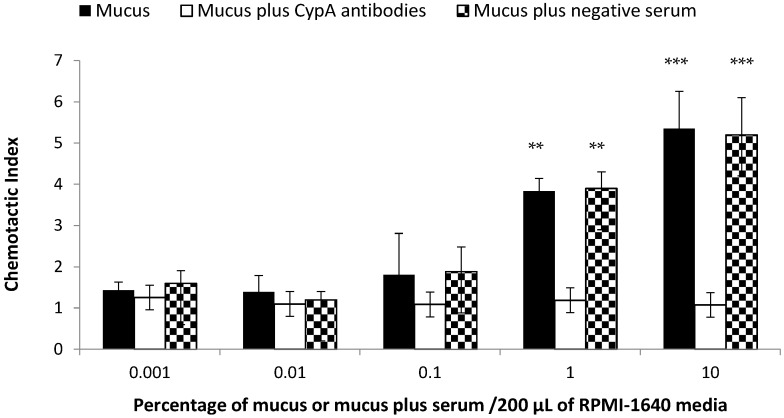
Comparative chemotactic activity of various concentrations of yellow catfish skin mucus. The black bar shows the chemotactic activity of various concentrations of yellow catfish skin mucus for head kidney leucocytes of yellow catfish. The white bar shows the chemotactic activity of the skin mucus when the CypA was blocked by CypA-specific antibodies. The doted bar indicates the chemotactic activity of skin mucus plus serum (negative control). The chemotactic activity is shown as the chemotactic index. Data are expressed as the means ± SD, (*n* = 3) of three fish. Bars with “**” are highly significantly different (*p* < 0.01), while bars with “***” are very highly significantly different (*p* < 0.001).

The chemotactic activity of various concentrations of the skin mucus is shown in Figure 2. The activity was very low when the concentration of mucus was 0.001% with the chemotactic index of 1.43, while it became significantly higher as the concentration of mucus raised to 1% and then 10% with chemotactic indexes of 3.83 and 5.35, respectively. Overall, the chemotaxis was strongly concentration-dependent as there were significant differences among the lower and higher and highest concentrations (Figure 2). However, the activity was significantly reduced after the addition of anti-CypA antibodies when compared with the negative control (rabbit serum plus skin mucus). After blocking CypA with antibodies in the mucus, the activity in the 0.001% group was 1.26, which was decreased gradually and was 1.18 and 1.07 in the 1% and 10% mucus concentration groups. However, the activity in the negative control group (rabbit serum plus mucus) was 1.6 in 0.001%, which rose to 3.9 in the 1% and 5.2 in the 10% concentration groups of skin mucus. All of these activities were significantly higher than the group treated with antibodies. This result suggests that the higher activity is due to CypA present in the mucus, as the rabbit serum has no effect on leukocyte migration (Figure 2).

### 2.3. Chemotactic Activity of Mutant CypA

**Figure 3:**
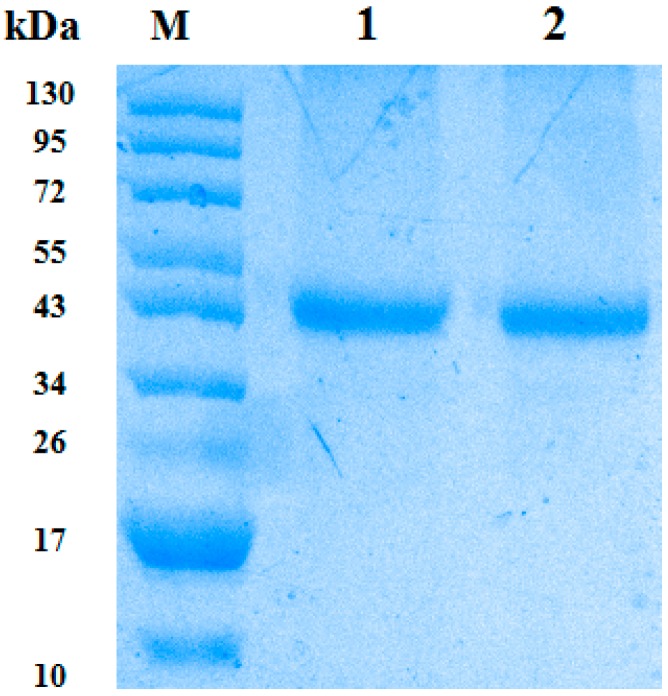
The expression and purification of GST-CypA. The CypA protein of yellow catfish was GST-fused expressed, purified and confirmed through SDS-PAGE, where the **M** represents the protein marker, **Lane 1** represents CypA^wt^ and **Lane 2** represents CypA^mt^ in this figure.

**Figure 4:**
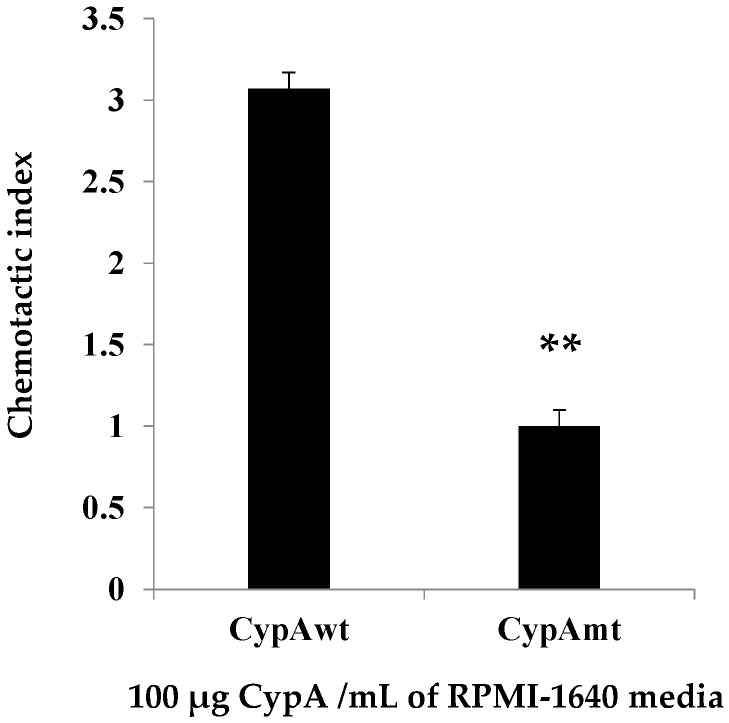
The chemotactic activity of recombinant CypA protein. The figure shows the comparative chemotactic activity of CypA^wt^ (wild type) and CypA^mt^ (mutant type) (100 µg/mL) of yellow catfish for head kidney leucocytes of yellow catfish. Data are expressed as the means ± SD, (*n* = 3) of three fish. The bar with “**” is highly significantly different (*p* < 0.01).

The wild-type and mutant-type CypA proteins (termed as CypA^wt^ and CypA^mt^) were purified after expression and are shown in Figure 3. Both of the GST-fused proteins have an equal size of 43 kDa, and their chemotactic activity is depicted in Figure 4. The chemotactic index was about 3.08 in wild-type, while it was one in CypA^mt^, which is significantly lower than wild-type. This significant lower chemotactic index in the CypA^mt^ confirms that Asn^69^ is responsible for chemotaxis.

## 3. Discussion

The body of knowledge about the role of fish skin mucus in host immunity compelled us to screen for its component characterization. Several studies demonstrated the presence of defensive proteins, including CypA, in the skin mucus of fish [10,12]. The skin mucus of yellow catfish has been recognized to have antimicrobial peptides, including pelteobagrin with strong anti-fungal and antibacterial activities [35]. Accordingly, in the present study, we have discovered interferon-induced GTP-binding protein Mx1, vasa short form, β-enolase, recombination activating protein 1, lsm12-like protein A and CypA from the skin mucus of yellow catfish by mass spectroscopy. These finding suggests that yellow catfish skin mucus is a strong defensive barrier against invading pathogens as CypA is associated in immune response against bacteria in yellow catfish [34], while enolase plays a role against cryptocaryoniasis [36]. Similarly, lsm12 is associated with translation regulation [37]; interferon-induced GTP-binding protein Mx1 has antiviral activity [38]; vasa play a key role in bovine spermatogenesis [39]; and recombination activating protein 1 has been considered as an immunological associate in fishes [40]. Our novel discovered proteins are addition to the previously reported immunological components, like immunoglobulins, agglutinins, lectins, lysins, lysozymes complement, carbonic anhydrase, crinotoxins, calmodulin, C-reactive protein, proteolytic enzymes and antimicrobial peptides in the fish skin mucus [41]. The number of proteins discovered in this study from the skin mucus of a healthy fish is less compared to those identified by others [42]. This interesting result suggests that fish can change the enzymes in the skin mucus according to environmental stimulus [43]. Nevertheless, together, these findings demonstrated the biochemically-rich nature of fish skin mucus.

CypA is ubiquitously expressed in all of the major tissues of teleost, including yellow catfish [34,44,45]. Accordingly, in the present study, we initially examined CypA by Western blot in the skin mucus of yellow catfish. Its expression detected by Western blot compelled us to examine its possible role in the skin mucus. Extracellular CypA can induce leucocyte migration in channel catfish and yellow catfish in in vitro examinations [33,34]. The fact that extracellular CypA attracts leucocytes in response to inflammatory stimuli is well documented [46,47]. In accordance with fish, the CypA was widely observed to encourage leucocyte migration in mammals [29,32,46,47,48,49]. In the present study, the secreted CypA in the skin mucus revealed strong chemotactic activity for yellow catfish leucocytes in a concentration-dependent manner. When CypA was blocked with anti-CypA antibodies inside the skin mucus, the chemotactic activity was decreased significantly compared to the negative control. This result suggests that the activity was solely due to CypA, as rabbit serum has no significant effect on chemotaxis. Our result hints that the fish secrete CypA in its mucus, which perhaps equips its epithelium by the infiltration of body leucocytes to fight against pathogens, because fish are permanently exposed to many pathogens, such as bacteria, viruses and parasites. To fight against these pathogens, fish uses the epidermis and its enzymatic secretion (mucus) [50], where it can change the clinical health parameters (e.g., enzymes) in the skin mucus according to environmental stimulus [43]. Moreover, the skin mucus of teleost fish is a strong chemoattractant for a variety of pathogenic bacteria [1,50,51]. Further studies could explore the precise molecular mechanisms involved in CypA chemotaxis and other possible roles in the skin mucus of fish.

Structurally, all CypA protein is composed of eight strands of antiparallel β-sheets in a flattened β-barrel with two helices capping the top and bottom. The residues Arg-55, Phe-60, Met-61, Gln-63, Phe-113, Trp-121, Leu-122 and His-126 constitute the PPIase active site. The residues Arg-55, Phe-60, Met-61, Gln-63, Gly-72, Ala-101, Asn-102, Ala-103, Gln-111, Phe-113, Trp-121, Leu-122 and His-126 constitute the CsA binding cite, while Arg-55 is the catalytic residue [52,53]. In mammalian CypA, Arg^69^, His^70^ and Thr^107^ are the critical residues responsible for leucocytes chemotaxis [54]. Hist^70^ and Thr^107^ are the conserved amino acids in the CypA of teleost (including yellow catfish). However, teleost fish CypA conserves Asn^69^ instead of Arg^69^. Whether the Asn^69^ residue is critical for chemotaxis in teleost CypA remains to be determined [33]. Therefore, we have proven that Asn^69^ is also the critical site responsible for chemotaxis in teleost CypA. As shown in the result, the chemotactic activity was significantly lowered (negligible) in CypA with the Asn^69^ mutant, compared to wild-type, indicating the precise residues for chemotaxis. This result provides an additional site (Asn^69^) critical for leucocyte chemotaxis in teleost fishes. Our result also hints at the high homology among teleost and mammals, as Arg^69^ was declared as the responsible site for chemotaxis in mammalian CypA [54]. Specifically, this discovery provides a new target site in teleost CypA, which would help in the development of anti-inflammatory drugs without altering the rest of their natural functions.

In conclusion, our mucus-screened map reveals the presence of multiple defensive proteins and suggests them being involved in host defense. The chemotaxis of CypA for leucocytes in the skin mucus provides novel evidence and suggests their wide association in the teleost immune and pathogenic processes. Our finding that residues Asn^69^ of yellow catfish CypA is essential for chemotaxis will help in the development of novel anti-inflammatory therapies, specifically for the inhibition of chemotaxis activity without affecting the PPIase activity of CypA. Further molecular-based studies are needed to explore the additional function of extracellular CypA (especially in the mucus), in order to fight against the wide range of inflammatory diseases.

## 4. Materials and Methods

### 4.1. Animals and Sample Collection

Mucus samples were collected from healthy yellow catfish (100–150 g, 6 fish/group), which were acclimatized for two weeks before the experiment, according to the method described previously [1]. Fish were anesthetized, and the lateral surface of the skin was gently rubbed to avoid contact with the epithelium, using a soft rubber spatula. The skin mucus was either immediately subjected to transwell for chemotaxis determination or immediately homogenized in 50 mM phosphate buffer solution (PBS), pH 6.0 containing 5 mM EDTA on ice by using a glass homogenizer. The insoluble substances were removed by centrifuging at 15,000× *g* for 30 min, and the supernatant was kept in a 1.5-mL sterile tube and stored in liquid nitrogen till further use. All experimental procedures involved in this study were permitted by the Institutional Animal Care and Institute of Huazhong Agricultural University (Ethical Approval No. HBAC20091138; Date: 15 November 2009).

### 4.2. Mass Spectrometric Analysis

Skin mucus was digested with sequencing-grade trypsin (Promega, Madison, WI, USA). MALDI-TOF MS and TOF/TOF tandem MS were performed on a MALDI-TOF-TOF mass spectrometer (4800 Proteomics Analyzer, Applied Biosystems, Foster City, CA, USA). Peptide mass fingerprints coupled with peptide fragmentation patterns were used to identify the protein in the International Protein Index (IPI) (http://www.ebi.ac.uk/IPI/IPIhelp.html) [55] database (Version 3.67) using online MASCOT search engine (http://www.matrixscience.com) [56]. This mass spectroscopic method for protein identification was also used by others [57] (data mining was performed in 2013).

### 4.3. Western Blot Analysis

Western blot analysis was performed to check the expression of CypA in the mucus samples. Our laboratory has previously synthesized antibodies for yellow catfish CypA [34], which were used for Western blotting. In order to quantify the CypA, skin mucus was subjected to 12% SDS-PAGE tailed by membrane transfer. Membranes were blocked for nonspecific binding with Odyssey blocking buffer (Li-Cor Biosciences, Lincoln, NE, USA) for 1 h at room temperature followed by incubation with anti-yellow catfish CypA rabbit serum diluted in Odyssey blocking buffer (1:10,000) overnight at 4 °C. Secondly, infrared dye-linked goat anti-rabbit IgG antibody (1:15,000) was added to the membranes and incubated at room temperature for 1 h. The results were visualized and quantified using an Odyssey infrared imaging system (Li-Cor Biosciences).

### 4.4. Sample Preparation, Plasmids Construction and Protein Expression

Mammalian CypA has 3 critical sites responsible for chemotaxis (His^70^, Thr^107^ and Arg^69^) [35]. Yellow catfish CypA conserves the 2 critical sites responsible for chemotaxis (His^70^and Thr^107^), but has Asn^69^ instead of Arg^69^. Whether Asn^69^ is also critical for chemotaxis needs scrutiny [33]. Therefore, the primers were designed (after the comparison of the yellow catfish CypA amino acid sequence with other vertebrates) for mutation in yellow catfish CypA and were based on that recommended site for chemotaxis determination (shown in Table 2). Total RNA was extracted from the liver of yellow catfish using RNAiso Plus (Takara, Dalian, China). For cDNA construction, the PrimeScript™ RT reagent kit with gDNA Eraser (Takara) was used following the manufacturer’s instructions. The liver cDNA was further used as templates for PCR amplification with degenerated primers. The PCR products were detected by 1.0% agarose gel electrophoresis, and the amplified DNA fragments were purified and ligated into pMD18-T simple vector (Takara) and used for sequencing. After confirmation of the desired sequence, the fragment was ligated in the expression plasmid.

The full length cDNA encoding yellow catfish-CypA was PCR amplified using the primers 5′-CTG **GGATCC** TAATGGCCAAACCCAGAGTGTT-3′ and 5′-CAG **CTCGAG** TTAAAGTTGGCCACAGTCAG-3′, in which BamH1 and XhoI restriction enzyme sites (bold and underlined) were added, respectively. After digestion with BamH1 and XhoI, the PCR product was ligated into the plasmid pGEX-5X-1, which produced proteins with the GST-tag. The recombinant plasmids CypA^wt^ (wild-type) and CypA^mt^ (Alan^69^-mutant) were transformed into *Escherichia coli* BL21 (DE3) competent cells (TransGen, Beijing, China), and the GST-fused CypA proteins were expressed. The induced recombinant proteins were purified using the Micro Protein PAGE Recovery kit (Sangon, Shanghai, China) following the manufacturer's instructions. The purified proteins were immediately checked for leucocyte migration in transwell plates (chemotaxis determination).

### 4.5. Leukocytes Separation

For the chemotaxis assay, leucocytes were isolated from the head kidneys of yellow catfish by the method described by Dong [34]. Briefly, fish were anesthetized with MS222 (Syndel, Nanaimo, BC, Canada); the head and body kidneys were aseptically removed and passed through a mesh (100 mm, Falcon) in RPMI-1640 containing 1% fetal bovine serum (FBS) (Gibco, Grand Island, NY, USA) and 200 IU/mL penicillin plus streptomycin (Amresco, Solon, OH, USA). The resulting cell suspension was layered onto a 34%/51% Percoll (Pharmacia, Stockholm, Sweden) density gradient and centrifuged at 400× *g* for 30 min at 4 °C. The interface was collected, and the cells were washed twice with RPMI-1640 at 400× *g* for 10 min at 4 °C before being resuspended to 1.35 × 10^6^ cells/mL inRPMI-1640 containing 1% FBS.

### 4.6. Chemotaxis Assay of Skin Mucus

To confirm the function of extracellular CypA present in the mucus, fresh skin mucus was checked for leucocytes migration. The transwell migration assay was used to measure the chemotactic activity of yellow catfish mucus that has an abundant amount of CypA. The transwell migration assay was performed with a 24-well costar transwell apparatus (Corning Costar Co., Cambridge, MA, USA). In the first phase, fresh mucus was diluted in RPMI-1640 medium plus 1% FBS to 0.001%, 0.01%, 0.1%, 1% and 10% (6.25, 12.5, 25, 50 and 100 µg/mL), and 600-µL aliquots of the dilutions were added to each lower chamber of the transwell units, except the control (blank). Subsequently, polycarbonate filters with a pore diameter of 8 µm were placed onto the lower wells, and 100 µL of target cells (1.35 × 10^6^ cells/mL) were added to the upper chamber. The unit was incubated on a shaker at 100 rpm (25 °C) for 90 min. At last, the number of cells that migrated into the lower chamber was counted under microscope, and the result was expressed as a chemotactic index (the number of cells that migrated in response to mucus divided by the number of cells that migrated to the RPMI-1640 medium plus 1% FBS (blank control)).

To check whether the chemotaxis is precisely due to CypA, we added antibodies in the second phase, and the above method and apparatus were used, except that anti-CypA anti-bodies were added (concentration 1:2000) to the lower chamber to block CypA inside the mucus, and the chemotactic activity was determined by the method as mentioned before. In this phase, the negative control group was also run, which contained rabbit serum plus mucus equal to the amount used in each group with antibodies.

### 4.7. Chemotaxis Assay of CypA Recombinant Protein

To explore and confirm the recommended residue responsible for leucocyte migration in yellow catfish CypA, we produced 2 types of protein (CypA^wt^ and CypA^mt^) as mentioned before. For the evaluation of chemotaxis, the same method (mentioned above for mucus) and apparatus were used for CypA protein chemotaxis with the exception that the CypA^wt^ and CypA^mt^ protein were diluted in RPMI-1640 medium plus 1% FBS to 100 µg/mL, and 600-mL aliquots of the dilutions were added to each lower chamber of the transwell units, except the control (blank). The number of cells that migrated into the lower chamber was counted under microscope, and the result was expressed as a chemotactic index (the number of cells that migrated in response to CypA^wt^ and CypA^mt^, divided by the number of cells that migrated to the RPMI-1640 medium plus 1% FBS (blank control)).

### 4.8. Statistics

The data are presented as the mean ± SD (*n* = 3). The data were analyzed using SPSS (20.0), and *p* < 0.05 was considered statistically significant.

## Figures and Tables

**Table 1:** Proteins putatively annotated in the skin mucus of yellow catfish using mass spectrometry. The detailed procedure used to putatively annotate the detected protein is present in the Materials and Methods section. Mucus was assessed by mass spectrometry; the identified proteins are shown with their molecular masses and the matching species with the accession number in the database.

Serial No.	Protein Name	*M*_W_ (Da)	Score	Species	Accession No.
1	Interferon-induced GTP-binding protein Mx1	72,882	30	*Ictalurus punctatus*	gi|82208280
2	Vasa short form	70,798	31	*Silurus meridionalis*	gi|188529679
3	β-Enolase	47,773	36	*Ictalurus furcatus*	gi|308321422
4	Recombination activating protein 1	46,987	28	*Pimelodus maculatus*	gi|382928308
5	lsm12–like protein A	21,644	31	*Ictalurus furcatus*	gi|308322011
6	Cyclophilin A	17,724	32	*Ictalurus punctatus*	gi|318264336

**Table 2:** Primers used for mutation in yellow catfish CypA. In both primers, the Asn amino acid is replaced by the Alan amino acid at position 69, which produced the CypA^mt^ protein. The codons for Alan (GCT and AGC) are presented bold in both forward and reverse primers.

Primer’s Name	Sequence (5′–3′)	Application
69: AAT-GCT (Forward)	GTGACTTCACA**GCT**CACAACG	cDNA amplification
69: AAT-GCT (Reverse)	GTG**AGC**TGTGAAGTCACCAC	cDNA amplification

## Supplementary Materials

Supplementary materials can be found at www.mdpi.com/1422-0067/17/9/1422/s1.
