# Supplementary material for: Chemotactic Activity of Cyclophilin A in the Skin Mucus of Yellow Catfish (Pelteobagrus fulvidraco) and Its Active Site for Chemotaxis
Source: Int J Mol Sci. 2016 Aug 29;17(9):1422. doi: 10.3390/ijms17091422 (PMC5037701; doi:10.3390/ijms17091422)
Supplement: Supplementary file 1 [file ijms-17-01422-s001.pdf]

Supplementary Materials: Chemotactic Activity of Cyclophilin A in the Skin Mucus of Yellow Catfish (*Pelteobagrus fulvidraco*) and Its Active Site for Chemotaxis

Farman Ullah Dawar, Jiagang Tu, Yang Xiong, Jiangfeng Lan, Xing Xing Dong, Xiaoling Liu, Muhammad Nasir Khan Khattak, Jie Mei and Li Lin

Table S1. Identified peptides against protein database. Peptide were identified using the protein in the International Protein Index (IPI) (http://www.ebi.ac.uk/IPI/IPIhelp.html) database using online MASCOT search engine. The peptides are presented with the reference number and percent identity which they showed with proteins in database.

Identified Peptides	Amino Acid Sequence	Reference Number	Identity
Interferon-induced GTP-binding protein Mx1	K.EISDLIRK.G<br>K.GQPENIGEQIK.R<br>K.EVNEYEEKYR.G<br>R.EMLQLMQDKNAIDHLLK.E<br>K.GFIQLAQNSFLGFPNLLKMAK.T<br>-.MSASLSEQYEEKVRPCIDLIDSLR.A	gi|82208280 (2013)<br>Q7T2P0 (2016)	85%
Vasa short form	R.EKALGDFR.T<br>K.LVNSPGMPPK.E<br>K.VFASTDTRR.G<br>MEDWEDDQSPVVTNTTFDNKESSWK.S	gi|188529679 (2013)<br>ACD62526 (2016)	100%
Beta-enolase	K.FSVVEQEK.I<br>R.IQQTVEKK.A<br>K.AGKYDLDFK.S<br>R.IEEELGDNAK.Y<br>K.FMLELDGTENK.S<br>K.FMLELDGTENKSK.F<br>R.SGETEDTFIADLVVGLCTGQIK.T	gi|308321422 (2013)<br>ADO27862 (2016)	100%
Recombination activating protein 1	R.GGGICSFTRTR.V<br>K.ENVEGAGRQVDLK.L<br>R.EPEVQGDLDESSR.Y<br>R.VPIWKPHNSHCLHCYPK.R	gi|382928308 (2013)<br>AFG29707 (2016)	100%
lsm12--like protein A	K.DCKWQEK.N<br>R.DLDQKSVQR.S<br>K.NIVVMDDVVITPPYR.A	gi|308322011 (2013)<br>ADO28143 (2016)	100%
Cyclophilin A	K.GFGYKGSGFHR.V<br>K.CFAKIVIADCGQL<br>K.HVVFGSVVDGMDVVR.V<br>K.SIYGNKFADENFTLK.H	gi|318264336 (2013)<br>NP_001187167 (2016)	100%
